# Finite element analysis of the angle range in trans-inferior alveolar nerve implantation at the mandibular second molar

**DOI:** 10.1186/s12903-023-03641-4

**Published:** 2023-11-25

**Authors:** Wenli Wu, Liangyue Song, Jinming Liu, Lingyi Du, Yuhang Zhang, Yingying Chen, Zichun Tang, Ming Shen

**Affiliations:** 1 Jiangsu Province Key Laboratory of Oral Diseases, Nanjing Medical University, Nanjing, 210029 China; 2 The Affiliated Stomatological Hospital of Soochow University, Suzhou Stomatological Hospital, Suzhou, 215000 China; 3 Jiangsu Province Engineering Research Center of Stomatological Translational Medicine, Nanjing Medical University, Nanjing, 210029 China; 4 Department of General Dentistry, Affiliated Hospital of Stomatology, Nanjing Medical University, Nanjing, 210029 China

**Keywords:** Trans- inferior alveolar nerve implants, Mandibular atrophy, Implant tilt angle, Mandibular second molar, Finite element analysis

## Abstract

**Background:**

Trans- inferior alveolar nerve (IAN) implantation technique was wildly used while the potential appropriate angle range in which the residual alveolar bone can bear the stress without absorption are currently unclear. This study aimed to evaluate the stress distribution pattern of the interface between bone and implant by finite element analysis (FEA) to determine the appropriate range of the implant tilt angle.

**Methods:**

Cone beam computed tomography (CBCT) images of 120 patients with missing mandibular second molars and vertical bone height < 9 mm in the edentulous area were selected. The distances from the mandibular nerve canal to the buccal cortex, the lingual cortex and the alveolar ridge crest were measured by using a combination of software. The angular ranges of the buccal-lingual inclination of simulated trans-IAN implants were measured and three-dimensional finite element models were constructed in the mandibular second molar area according to the differences of the inclination angles. A vertical load (200N) was then applied to analyze the biomechanical conditions of the implant-bone interface during median occlusion.

**Results:**

The distance at the second molar from the nerve canal to the buccal cortex, lingual cortex and alveolar crest were 6.861 ± 1.194 mm, 2.843 ± 0.933 mm and 7.944 ± 0.77 mm. Trans-IAN implantation was feasible in 73.33% of patients. The minimum angle and maximum angles of the buccal-lingual inclination of the simulated implant were 19.135 ± 6.721° and 39.282 ± 6.581°. When a vertical static load of 200N was applied, the tensile stress in cortical bone gradually increased with the increase of the implant tilt angle. When the inclination angle reached 30°, the tensile stress (105.9 MPa) exceeded the yield strength (104 MPa) of cortical bone. Compared with the conventional implants, the stress peak value of the vertical ultra-short implant in cortical bone was greater than the stress peak value of the conventional implants at 10°(79.81 MPa) and 20°(82.83 MPa) and was smaller than the stress of the implant at 30°(105.9 MPa) and 40°(107.8 MPa). Therefore, when the bone mass allows, conventional-length implants should be selected whenever possible, and an operative range of the trans-IAN implantation in the mandibular second molar could be retained with an inclination angle of < 30°.

**Conclusions:**

The mandibular nerve canal at the mandibular second molar was obviously biased to the lingual side, which ensured sufficient bone mass at the buccal side. In most patients with severe mandibular atrophy, it was possible to maintain a safe distance from the nerve canal with conventional-length implants via the trans-IAN implantation technique.

## Introduction

Adequate bone is necessary for dental implant reconstruction. However, periodontitis, prolonged edentulous status, prolonged use of removable dentures and other factors can cause extensive absorption of the alveolar bone, and dental implantation is often limited by anatomical conditions including jaw morphology and the location of the maxillary sinus and the inferior alveolar nerve (IAN) canal [1–3]. The insufficient bone mass caused by excessive gasification of maxillary sinus can be increased by maxillary sinus floor elevation [4]. And for the mandible, the nerve canal especially limits the amount of available bone in the posterior mandible. Methods including guided bone regeneration, autologous or allogeneic bone transplantation, distraction osteogenesis and others can increase the available bone [5, 6], but the risk of complications, including postoperative infection, also increases [7–9]. If the existing bone mass can be used for a better implant restoration without additional surgical procedures, the risk of such complications can be reduced. The use of short (6 to 8 mm) [10] or ultrashort (≤ 6 mm) [11] implants is one option for avoiding incremental bone surgery in patients with severe mandibular atrophy that precludes vertical implantation of conventional implants [12], but short implants have generally shown poorer stability and safety when compared with conventional implants. Therefore, when the bone mass allows, conventional-length implants should be chosen whenever possible, and during implant reconstruction of the posterior mandibular teeth, the length and diameter of the implant should be maximized and the existing bone height should be utilized to the greatest extent possible.

Cone beam computed tomography (CBCT) is a 3D x-ray imaging technology that overcomes the limitations of traditional two-dimensional dental imaging, and can more accurately depict facial bone structure and surrounding soft tissues [13]. Previous studies examining the orientation of the mandibular nerve canal by using CBCT have shown that from the first premolar to the third molar, the nerve canal gradually moves from the buccal side to the lingual side [14]. As a result, under the condition of ensuring a safe distance of 1.5 mm between the edge of the implant and the inferior alveolar nerve (IAN) [15], a trans-IAN implantation technique that makes full use of the bone mass on the buccal or lingual side of the mandibular canal has been introduced [16]. In this study, firstly, the anatomical position of the mandibular canal was measured by using CBCT in 120 patients with missing mandibular second molars and severe vertical bone mass shortage. Then, trans-IAN implantation was simulated in a proprietary simulator (SIMPLANT Pro 17.01, Leuven, Belgium), and the implantation angle was measured. Three-dimensional finite element analysis is an efficient, noninvasive, accurate and repeatable biomechanical research method ideally simulating complex conditions. It has been widely used in dental implant prosthetics since it can analyze and describe the biomechanical behavior of implant-supported dentures under occlusal force through accurate modeling and algorithm [17]. Hence a finite element analysis (FEA) for the trans-IAN implants was performed within this angle range. A previous retrospective analysis of implants that were inclined at a certain angle to the alveolar ridge bone surface by Krekmanov et al. has verified the good clinical effect of tilted implants [18]. However, the physiological limits of the amount of stress the alveolar bone can bear while maintaining its structure and strength without absorption are currently unclear. Therefore, in this study, under the condition of median occlusion, mandibular second molar region implantation was simulated with different angles of buccal tilt, and the finite element method was used to analyze the stress on the bone tissue around the implant to explore the appropriate range of tilt angles for the implant in order to provide a theoretical reference for clinical application design.

## Materials and methods

### Medical records and inclusion criteria

A total of 2458 records from patients with missing mandibular second molars who underwent pre-implant imaging in the Stomatological Hospital Affiliated Nanjing Medical University from January 2019 to March 2022 were screened by CBCT data, and 120 patients in whom short implants could not be vertically implanted [9] were selected for the study according to the inclusion criteria and analyzed by using the CBCT data [19]. The inclusion criteria were (1) age 20 to 70 years with missing second molars on either side of the mandible; (2) distance between the mandibular nerve canal and the alveolar crest in the mandibular second molar space < 9 mm. The exclusion criteria were (1) distance between mandibular nerve canal and the alveolar crest in mandibular second molar space ≥ 9 mm; (2) all teeth in the quadrant missing; (3) history of head and neck trauma, tumor or congenital malformation; (4) CBCT unclear.

### Instruments and equipment

Imaging was performed by CBCT (NewTom, QR srl, Verona, Italy) and three-dimensional (3D) image reconstruction and data measurement were performed with the proprietary software (SIMPLANT Pro 17.01, Leuven, Belgium). The establishment of the finite element model required the combined application of multiple software packages, including (Pro/Engineer 2001; PTC, Needham, Mass), Mimics 21 (Material, Belgium), Geomagic Studio 2022 (Geomagic, US), SolidWorks 2022 (Dassault Systems S.A, France), HyperMesh 2021(Altair, USA) and ABAQUS CAE 2021 (SIMULIA, USA).

### Image measurements

**Fig. 1:**
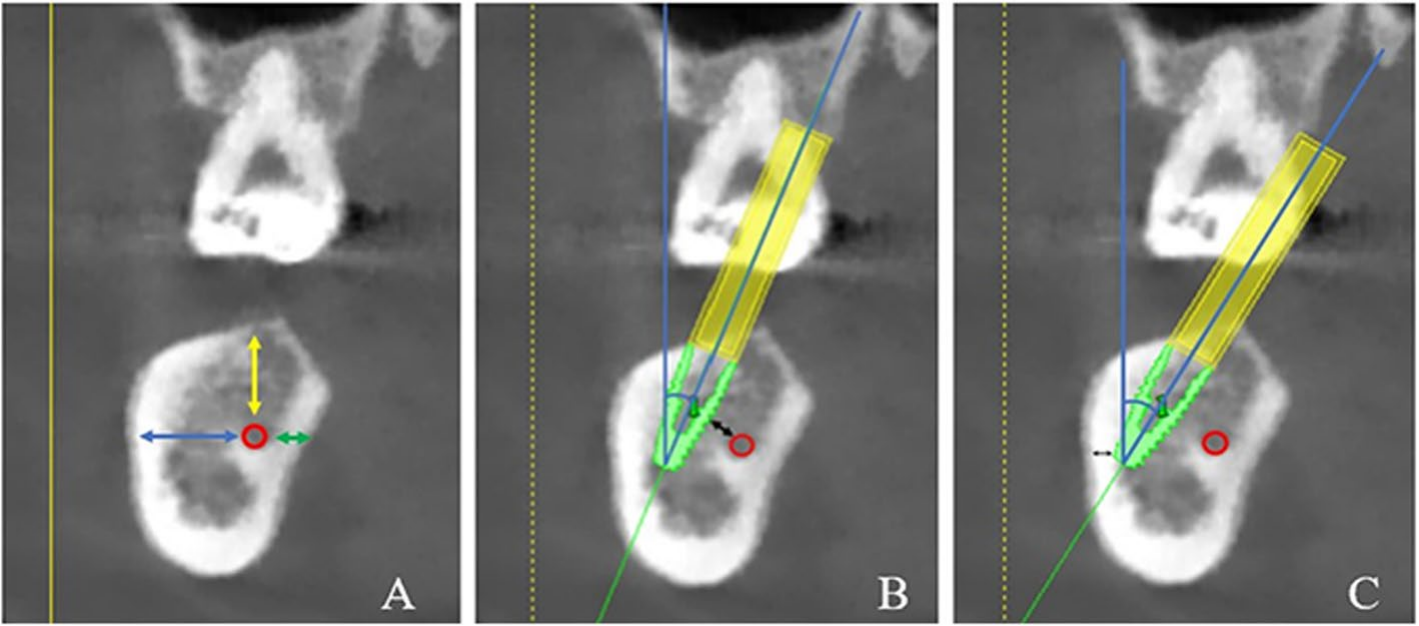
Cone beam computed tomography. **A** Measurement of the distance from the mandibular nerve canal (red circle) to the buccal cortex (blue arrow), the lingual cortex (green arrow) and the alveolar crest (yellow arrow). **B** and **C** Measurement of the minimum (**B**) and maximum (**C**) angles of buccal-lingual inclination of the virtual implant

First, the head position on CBCT was corrected from the coronal, sagittal and horizontal planes for each patient by using the Simplant software. Curved sections were then reconstructed along the line connecting the midpoint of the mandibular alveolar ridge. The distances from the mandibular canal to the buccal cortex, lingual cortex and alveolar crest were measured in a vertical cross-section at the center of the mandibular second molar space on the curved surface (Fig. 1A). If multiple consecutive teeth in the mandible were missing, the vertical cross-section at the midpoint of the mesial-apical line of the maxillary second molar was taken for measurement. All patients were virtually implanted with 4.1 × 10-mm (Bone Level Tapered Roxolid SLA, Straumann, Switzerland) to analyze the feasibility of the simulated trans-IAN implantation and to further screen out the patients in whom the ultrashort implant could not be vertically implanted for the feasibility simulation. A safe distance of at least 1.5 mm from the mandibular canal and the buccal bone plate was maintained and the minimum and maximum buccal-lingual inclination angles of the implant were measured (Fig. 1B, C).

### Establishment of the finite element model

As described above, in this study, virtual implants were implanted at the second molars of 120 patients and the minimum and maximum angles of buccal-lingual inclination of the implants were measured. This range of angles provided a preliminary analysis from a spatial aspect of the feasibility of the trans-IAN implantation. Next, the stress distribution at the bone interface of the implant under different inclination angles was analyzed by a 3D finite element method, and the safe inclination angle of the implant was further determined from the perspective of biomechanics.

**Fig. 2:**
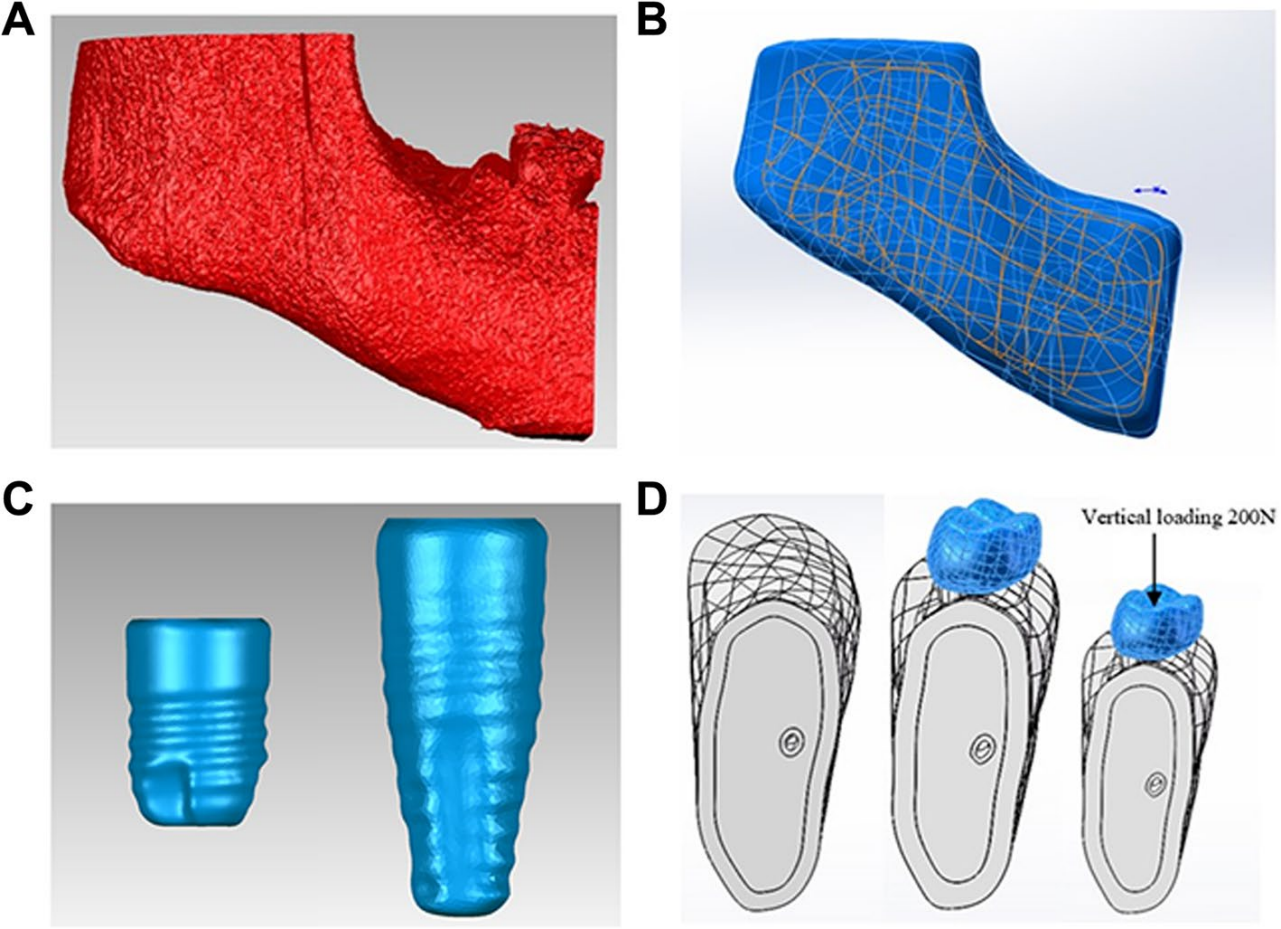
Model for the finite element analysis. **A** The right lower posterior dental bone mass comprising the mandibular first molar. **B** The mandible with cortical bone thickness of approximately 2 mm trimmed by multi-software to include only the mandibular second molar. **C** The two implant types evaluated in this study, left: ultra-short implant (4.0 × 6-mm), right: conventional implant (4.1 × 10-mm). **D** A portion of the second molar region was excised as the final mandibular model and the implant and prosthetic crown were installed

Mandibular CBCT data from a representative patient was selected from the 120 included cases and processed using multi-software (Mimics 21, Geomagic Studio 2022) to establish a mandible model containing the mandibular nerve canal (Fig. 2A). The mandible model was set as cortical bone with surface thickness of approximately 2.0 mm [20–22] and cancellous bone at the inside, and the second molar region was intercepted as the final mandible model (Fig. 2B). A 3D CAD model of the implant and abutment was then created using solid modeling software (Pro/Engineer 2001; PTC, Needham, Mass) with 4.1 × 10-mm implant (Bone Level Tapered Roxolid SLA, Straumann, Switzerland) and 4.0 × 6-mm (OsseoSpeed TX 4.00S, Astra Tech system, Dentsply Sirona, USA) (Fig. 2C). The main purpose of the short implant was to explore the feasibility of trans-IAN implantation when alveolar atrophy prohibited the vertical placement of a short mandibular second molar implant.

At this point, based on the range of implant inclination angles obtained with the imaging simulation, we established five models to examine the (1) vertical implantation of an ultra-short implant (0°) and (2–5) the buccal inclinations of conventional implants of 10°, 20°, 30° and 40°. In model 1, the normal line of the curved surface of the upper half of the mandible was taken as the baseline, and the baseline for the 0° implant was determined along this direction. The mandible, the ultrashort implant and the prosthetic crown were assembled using SolidWorks 2022, thereby obtaining the overall model for the 0° implant. For the subsequent models, the normal line of the curved surface of the upper half of the mandible was taken as the baseline, which was the line of intersection of the buccal-lingual plane. By using the intersection point of the curved surface and its normal line of the upper half of the mandible as the starting point and then in the buccal-lingual plane, according to intervals of 10°, we made the installation midline of the oblique implant toward the buccal side. The mandible, implant and crown were assembled at different angles of implantation. Thus, models with the implant tilted at 10°, 20°, 30° or 40° on the buccal side were obtained (Fig. 2D).

The convergence test was used to refine meshes until the change was less than 5% [20, 23, 24], and the number of elements was increased to obtain accurate results in this study. The mesh size was 0.3 mm near bone-to-implant contact and differed in other areas. For the five configurations, the number of elements was 1,147,430; 1,223,549; 1,178,033; 1,232,213 and 1,264,386, and the number of nodes was 216,409; 230,236; 222,429; 233,114 and 239,238, respectively.

Data from the meshed models were transferred to a finite element analysis program (ABAQUS CAE 2021) for preprocessing procedures. All models were homogeneous, linear elastic and isotropic. The mechanical properties (Young's modulus and Poisson’s ratio) are shown in Table 1. The implant and bone interface are defined as the “tie” for complete bone combination. The mandible was completely constrained in the mesial and distal directions to simulate the actual situation of the mandible. In this way, after the masticatory force was loaded, the model would not undergo overall displacement, but the bone tissue in the constrained area could undergo deformation and displacement. An average occlusal force of 200N [21, 22, 25] was used and the implant was subjected to the vertical load through the crown.

**Table 1:** Mechanical properties of the finite element model components

Material	Young’s modulus (MPa)	Poisson’s ratio	Reference
Cortical Bone	13,700	0.3	[21]
Cancellous Bone	1370	0.3	[26]
Titanium alloy	110,000	0.34	[21]
Crown(zirconia)	210,000	0.35	[27]
Nerve Canal	70	0.45	[21]

### Statistical methods

The results were measured by the same researcher once every two weeks. SPSS 19.0 was used to analyze the consistency of the two measurements by intraclass correlation efficient (ICC). After the high consistency of the two results was confirmed, the average value of the two results was taken to calculate the average value and standard deviation, and the difference was analyzed by *t* test. The FEA was carried out by using ABAQUS CAE 2021 software. Considering that implants and restorations are made of malleable materials, the von Mises stress analysis was adopted [27, 28]. However, bone tissue is brittle (non-extensible material). In order to better understand the influence of different inclination angles of implants on the stress distribution in bone tissue around the implants, the maximum principal stress (tensile stress) and the minimum principal stress (compressive stress) were also obtained [29–31].

## Results

### Gender differences in the anatomical position of the mandibular nerve canal

The study included imaging data for 45 male patients and 75 female patients. The distances between the mandibular nerve canal and buccal cortex, lingual cortex and alveolar crest at the second molar were 6.861 ± 1.194 mm, 2.843 ± 0.933 mm and 7.944 ± 0.770 mm. The distance from the mandibular nerve canal and the buccal cortex was significantly longer than that from the canal to the lingual cortex in both male and female patients, but there was no significant difference between genders in the distances between the mandibular nerve canal and the three sites (Table 2).

**Table 2:** Comparison by gender of the distance between the mandibular nerve canal and the buccal cortex, lingual cortex and alveolar crest in the edentulous mandibular second molar space with alveolar bone height < 9 mm The t1, p1 and Cohen’s d are the between-gender differences in the distance from the mandibular nerve canal to the buccal cortex, lingual cortex and alveolar crest; t2, p2 and Cohen’s d are the differences in the distance from the mandibular nerve canal and the buccal cortex and lingual cortex among male patients, female patients and all patients

Gender	n	Buccal cortex/mm	Lingual cortex/mm	Alveolar crest/mm	t2	p2	Cohen’s d
Male	45	6.895 ± 1.143	2.738 ± 1.044	8.055 ± 0.824	18.016	0.000***	3.788
Female	75	6.841 ± 1.232	2.905 ± 0.861	7.957 ± 0.738	22.685	0.000***	3.704
Total	120	6.861 ± 1.194	2.843 ± 0.933	7.944 ± 0.770	29.047	0.000***	3.750
t1		0.238	-0.950	0.671			
p1		0.812	0.344	0.503			
Cohen’s d		0.045	0.179	0.127			

***P<0.001

### Feasibility of the simulated trans-inferior alveolar nerve implantation

Simulated trans-IAN implantation was possible in 88 of 120 patients (73.33%), including 33 of the 45 male patients (73.33%) and 55 of the 75 female patients (73.33%). Subsequently, we further analyzed a total of 26 cases in which the ultra-short implant could not be vertically implanted in the mandibular second molar, that is, the height of the alveolar ridge was < 7.5 mm. Among them, 12 patients (46.15%) had the possibility of theoretically inserting inferior alveolar nerve (Table 3).

**Table 3:** Simulation of trans-inferior alveolar nerve implant (ultra-short vertical prosthesis) in patients with missing mandibular second molar and alveolar bone height < 7.5 mm

Gender	Number of samples where 4.1*10 mm implants could be placed	Total sample size	Percentage
Male	2	7	28.57%
Female	10	19	52.63%
Total	12	26	46.15%

### Minimum and maximum buccal-lingual inclination angles of implants in simulated trans-inferior alveolar nerve implantation

A total of 88 patients who were eligible for trans-IAN implantation were analyzed. The minimum buccal-lingual inclination angle of the implant was 19.135 ± 6.721° and the maximum inclination angle was 39.282 ± 6.581°. The between-gender difference of the inclination angles of the simulated implant was not statistically significant (Table 4).

**Table 4:** Comparison by gender of the buccolingual inclination of simulated trans-inferior alveolar nerve implants in the edentulous mandibular second molar space with alveolar bone height < 9 mm

Gender	*n*	Minimum angle/ (°)	Maximum angle/ (°)
Male	33	18.418 ± 7.806	39.176 ± 7.663
Female	55	19.566 ± 6.013	39.345 ± 5.912
Total	88	19.135 ± 6.721	39.282 ± 6.581
t		-0.774	-0.191
p		0.441	0.849
Cohen’s d		0.17	0.026

### Stress distribution of implant

**Fig. 3:**
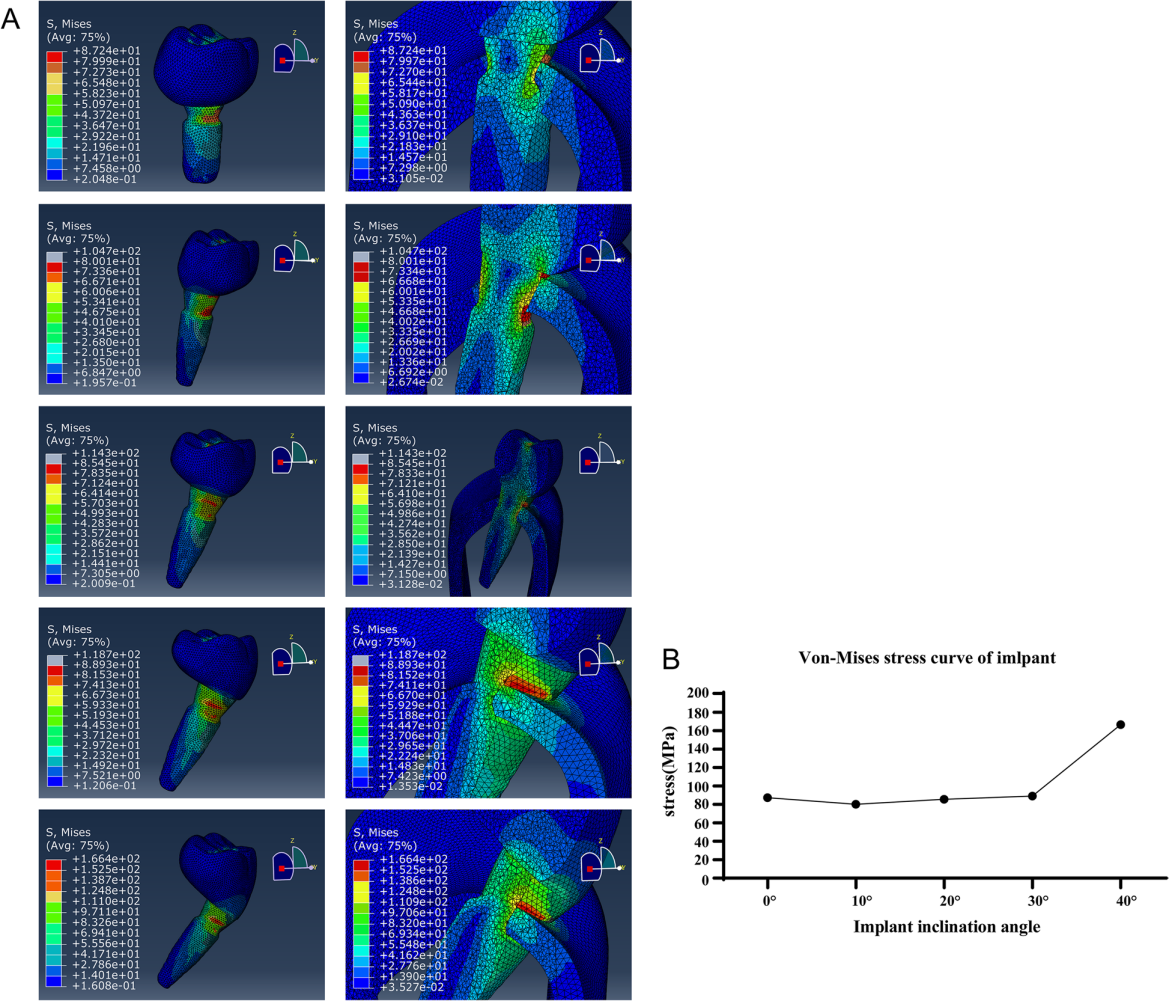
The nephogram (**A**) and graph (**B**) of the von Mises stresses for all configurations under vertical loading. **A** The von Mises stress concentrations on implant components in all configurations. The conventional implants with inclination angles of 0°, 10°, 20°, 30° or 40° respectively from left to right and top to bottom. **B** Stress curve of the implants under different inclination angles

Figure 3 shows the von Mises stress nephogram and graph of the implant under vertical static load. The stress peak was concentrated in the proximal neck of the implant for both the vertically placed ultra-short implants and the obliquely implanted conventional implants. Moreover, when using the conventional implant for the trans-IAN implantation, the von Mises stress of the implant increased with the increase of the inclination angle. The minimum von Mises stress was 80.01 MPa and occurred when the buccal-lingual inclination was 10° and the maximum von-mises stress was 133.6 MPa and occurred when the buccal-lingual inclination was 40°. Compared with the conventional implants, the stress peak value of the vertical ultra-short implant was 87.24 MPa, which was greater than the stress peak value of the conventional implants at 10° (80.01 MPa) and 20° (85.45 MPa) and was smaller than the von-mises stress of the implant at 30°(88.93 MPa) and 40°(166.4 MPa). All the stress peak value under these conditions far away from the yield strength of the implant (800 MPa) [31–33]. The stress peak was concentrated in the proximal neck of the implant, and it increased with the increase of the inclination angle.

### Stress distribution at the implant-bone interface (cortical bone)

**Fig. 4:**
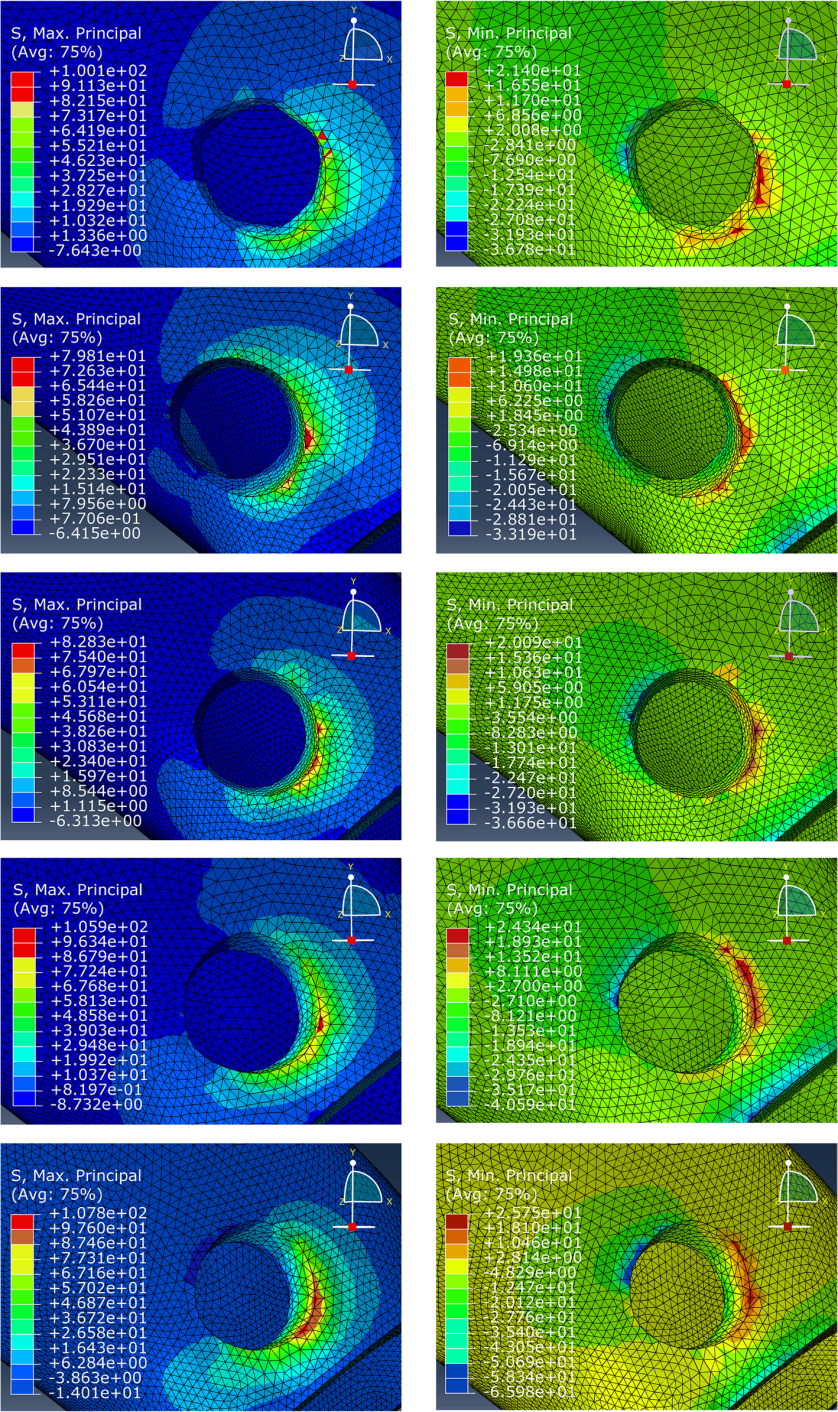
Left: Magnified view of tensile (maximum principal) stress of peri-implant cortical bone for short implant axial implantation and conventional implant tilt 10° 20° 30° 40°implantation from top to bottom; Right: Magnified view of compressive (minimum principal) stress of peri-implant cortical bone for short implant axial implantation and conventional implant tilt 10° 20° 30° 40° implantation from top to bottom

**Fig. 5:**
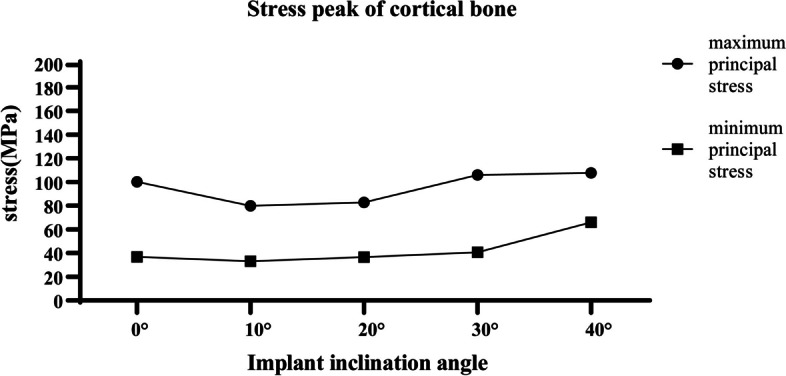
Maximum principal stress and minimum principal stress for all configurations under vertical loading

The maximum principal stress and minimum principal stress in the cortical bone were analyzed by the finite element software (Fig. 4). The maximum principal stress refers to the tensile stress and the minimum principal stress refers to the compressive stress. Figure 4 shows that the tensile stress was greater than the compressive stress in all five models. When the implant was tilted by 10°, the tensile stress was the lowest (79.81 MPa). The stress peak was located in the cortical bone around the proximal and middle neck of the implant. The tensile stress gradually increased with the increasing inclination angles. The tensile stress of cortical bone reached 82.23 MPa, 105.9 MPa, 107.8 MPa when the inclination angle was 20°, 30°, 40° respectively, but the position of the stress peak did not change significantly across the models. Previous studies have shown that the yield strength of the cortex is 104 MPa and 169 MPa under tension and compression respectively. Therefore, when the tilt angle reaches 30°, there is a risk of failure of the clinical trans-IAN implant. Compared with the oblique implants, the tensile stress value of cortical bone (100.1 MPa) when the ultra-short implant was vertically implanted was less than that of the implant with an inclination angle of 30° (Fig. 5), which does not exceed the yield strength of cortical bone. The range of variation of the minimum principal stress was not large in the five models, and all of the values were much smaller than the yield strength of cortical bone. The minimum principal stress peak was also located in the cortical bone around the middle neck of the implant. The stress peak was located in the cortical bone around the proximal and middle neck of the implant, and when the inclination angle reached 30°, the stress peak exceeded the yield strength of the cortical bone.

## Discussion

The mandibular nerve canal contains the inferior alveolar nerve, artery and vein. It is an important anatomical structure of the mandible that must be protected during implantation procedures. Previous studies have shown that from the first premolar to the third molar, the mandibular nerve canal gradually moves from the buccal side to the lingual side [14], which means trans-IAN implantation mostly concerns the area of the mandibular second molar. In this study, we measured and analyzed the location of the mandibular nerve canal in a group of patients with missing mandibular second molars and severe alveolar bone atrophy. The bone width of 6.861 ± 1.194 mm on the buccal side of the canal at the mandibular second molar was significantly larger than that of the lingual side. Therefore, in this study, a simulated trans-IAN implantation was performed (SIMPLANT Pro 17.01, Leuven, Belgium), and the maximum and minimum inclination angles of the implant were measured by virtual implantation in the region of the mandibular second molar. We found that the minimum inclination angle of the implantable buccal-lingual direction was 19.135 ± 6.721° and the maximum inclination angle was 39.282 ± 6.581°. In addition, in nearly a quarter of the 120 patients in this study, the distance from the mandibular nerve canal to the crest of the alveolar ridge was < 7.5 mm, which means that vertical implantation of even a 6-mm ultra-short implant was not possible in these patients [11]. However, in this subgroup, up to 73.33% of patients were candidates for implantation of a 4.1 × 10-mm cone-shaped implant via trans-IAN implantation, indicating better prospects for application of this technique. Based on these results, we used 3D FEA to further analyze the feasibility of trans-IAN implantation in the mandibular second molar region from the perspective of biomechanics [34–36] in order to explore various tilt angles and to provide a theoretical basis for clinical practice.

It is generally believed that the masticatory force is transmitted to the bone tissue through the implant, and appropriate stress stimulation can not only prevent disuse atrophy around the implant, but also facilitate the growth and remodeling of bone, promoting the formation of bone union [37] and maintaining continuous stability of the implant. Excessive stress will cause absorption and necrosis of bone tissue [38, 39]. However, in the case of inclined implantation, how much stress the alveolar bone bears can maintain the structure and strength of the bone without causing damage and absorption [33, 37], and the degree of inclination is still unclear [36, 40]. In all models of this study, the stress concentration is in the neck and the junction of the implant, which is consistent with other previous study on inclined implants [41]. Hamed et al. analyzed the stress at the bone interface of a single implant at different inclination angles in the maxillary posterior teeth area by three-dimensional finite element method. It was found that the oblique implant with an implantation angle of 25 degrees produced 66% more cortical bone stress than the vertical implant, and the stress of the implant exceeded 15% to 70% compared with the vertical implant [42]. However, there is little research and discussion on biomechanics of implant inclining at different angles when the mandibular molar area is implanted with inferior alveolar nerve. In this study, the stress at the bone interface of a single implant with different inclination angles was analyzed. When a vertical static load of 200N was applied, the tensile stress in cortical bone gradually increased with the increase of the implant tilt angle. When the inclination angle reached 30°, the tensile stress (105.9 MPa) exceeded the yield strength (104 MPa) of cortical bone [31–33]. Compared with the conventional implants, the stress peak value of the vertical ultra-short implant was 87.24 MPa, which was greater than the stress peak value of the conventional implants at 10° (80.01 MPa) and 20° (85.45 MPa) and was smaller than the von-mises stress of the implant at 30°(88.93 MPa) and 40°(166.4 MPa). Short implants are known to have higher variability and lower predictability of survival, with a 29% increased risk of failure compared to conventional implants in the posterior mandibular region [43–45]. Therefore, when the bone mass allows, conventional-length implants should be selected whenever possible, and an operative range of the trans-inferior alveolar nerve implant with an inclination of < 30° can be preserved for most patients with obviously insufficient bone mass in the posterior mandibular region.

Similar to trans-IAN, transposition of the IAN avoids the need for bone augmentation. This technique, which is mostly used for implantation of multiple consecutive missing teeth, requires moving the inferior alveolar nerve and blood vessels out buccally from the mandibular nerve canal so that the implant can pass through the canal without damaging the nerve and blood vessels [46]. The IAN is reset after the implant is placed. However, although this method can prevent damage to the IAN, the direct instrumentation at the mandibular nerve canal, the bias to the lingual side of the canal and the removal of more bone on the buccal side at the position of the mandibular second molar can lead to increased risk of postoperative swelling, infection and even jaw fracture [47, 48], and this approach has no obvious advantage in terms of technical difficulty and postoperative complications.

With the development of digital dental technology, trans-IAN implantation is supported in terms of safety and convenience, and a computer-aided dynamic navigation system (CADNS) based on CBCT is now available for implantation procedures. CADNS offers good predictability, accuracy and low risk compared to traditional implant surgery [49–51]. In a recent case report, a 4.1 × 10-mm implant was successfully placed by trans-IAN implantation in the mandibular second molar area under CADNS in a patient with a distance of only 4.5 mm between the alveolar ridge and the mandibular nerve canal. This implantation kept a safe distance of 1.7 mm between the implant and the nerve canal, and there was stable marginal bone and good bone union at the 6-month follow-up. This demonstrates the feasibility, safety and convenience of trans-IAN implantation in these cases.

In our study, the structures in the model were assumed to be homogeneous, isotropic and lineal elastic, and the implant-bone interface was assumed to exhibit complete osseointegration [33]. Therefore, the calculated results may be different from the clinical actual situation to some extent, and further in-depth study and clinical verification are required.

## Conclusion

For patients with missing mandibular second molars and significant alveolar bone atrophy, the relatively simple and safe technique of trans-IAN implantation, rather than complex bone augmentation, can be used in most cases because of the apparent proximity of the mandibular canal to the lingual cortical bone. When using trans-IAN implantation, a conventional-length implant should be selected as often as possible so that an operative range of inclination within 30° can be preserved. The use of CBCT-based CADNS can markedly increase the accuracy of the surgery.

## Data Availability

The datasets used and analysed during the current study available from the corresponding author on reasonable request.
